# Risk Factors for Emergency Department Presentations after the Initiation of Opioid Analgesics in Non-Cancer Patients in Korea: A Nationwide Study

**DOI:** 10.3390/medicina59030519

**Published:** 2023-03-07

**Authors:** Yoojin Noh, Kyu-Nam Heo, Dal-ah Kim, Ji-Min Han, Ju-Yeun Lee, Young-Mi Ah

**Affiliations:** 1 Pharmacy School, Massachusetts College of Pharmacy and Health Sciences, 19 Foster St., Worcester, MA 01608, USA; 2 College of Pharmacy and Research Institute of Pharmaceutical Sciences, Seoul National University, Seoul 08826, Republic of Korea; 3 Department of Anesthesiology and Pain Medicine, Incheon St. Mary’s Hospital, College of Medicine, The Catholic University of Korea, Incheon 22711, Republic of Korea; 4 College of Pharmacy, Chungbuk National University, Cheongju 28160, Republic of Korea; 5 College of Pharmacy, Yeungnam University, Gyeungsan 35841, Republic of Korea

**Keywords:** opioid analgesics, emergency department, national claim database, adverse drug events

## Abstract

*Background and Objectives*: Opioid use in Korea is lower than in other developed countries. However, recent studies have reported an increase in opioid prescriptions and the number of chronic opioid users. The current status of adverse events (AEs) associated with opioid analgesics in Korea is unclear. This nested case–control study aimed to evaluate the influence of opioid analgesic use patterns on all emergency department (ED) visits and opioid-related ED visits after opioid analgesic initiation using the national claims database. *Materials and Methods:* Adult non-cancer patients who initiated non-injectable opioid analgesics (NIOA) between January 2017 and June 2018 were included. We defined the case group as patients who visited the ED within six months of opioid initiation, and the control group was selected in a 1:1 ratio using an exact matching method. *Results:* A total of 97,735 patients (13.58%) visited the ED within six months of NIOA initiation. Nearly 32% of cases were linked to opioid-related AEs. The most frequent AEs were falls and fractures (61.27%). After adjusting for covariates, opioid initiation at the ED was associated with all-cause or opioid-related ED visits (adjusted odds ratio (aOR) = 3.19, 95% confidence interval (CI) = 3.09–3.29; aOR = 3.82, 95% CI = 3.62–4.04, respectively). Chronic NIOA use was associated with all-cause and opioid-related ED visits (aOR = 1.32, 95% CI = 1.23–1.40; aOR = 1.56, 95% CI = 1.39–1.76, respectively). *Conclusion:* This study found that 13% of non-cancer patients visited the ED within six months of NIOA initiation. In addition, the NIOA use pattern was significantly associated with all-cause and opioid-related ED visits.

## 1. Introduction

Prescription opioid analgesics (opioids) are commonly used for chronic non-cancer pain management, despite limited evidence regarding their safety and efficacy. Indeed, the use of opioids has been reported to be associated with many issues, including misuse, overuse, and other adverse effects [1,2]. Over the past two decades, we have witnessed a dramatic rise in opioid use, with many unintended consequences, such as opioid overdose, misuse, related utilization of hospitals, and even death, particularly in North America [3,4,5]. According to these negative consequences of opioid use, the United States government declared an opioid crisis and introduced various strategies to regulate opioid prescriptions, including prescription drug monitoring programs, lock-in programs, and opioid rescheduling [6,7,8]. The number of deaths related to prescription opioids has decreased (17,029 in 2017 vs. 14,139 in 2019) due to these various efforts, but the opioid crisis is still ongoing [9].

Emergency department (ED) visits could be an indicator of medication-related adverse events (AEs), causing additional hospital visits or emergency hospitalization in outpatient settings. Thus, the Institute for Healthcare Improvement recommended ED visits as a trigger tool to detect medication-related AEs in outpatient settings [10]. In studies on the adverse drug events (ADEs) associated with opioids, several previous studies used ED data [5,11], and ED visit monitoring was recommended as a surveillance method in the National Action Plan for Adverse Drug Events Prevention in the United States for opioid safety [12]. In contrast to the decrease in overdose death associated with prescribed opioids, the rate of ED visits related to opioid-related harm has plateaued since 2016 (242 per 100,000 population), despite various efforts to combat opioid use [13]. The United States authorities have recently emphasized efforts to reduce opioid-related ED visits [14].

In Korea, opioid use is lower than that in other developed countries. However, recent studies have reported an increase in the number of opioid prescriptions (1.52-fold increase from 2009 to 2019) [15], as well as an increase in the number of chronic opioid users (6-fold increase in chronic strong opioid users from 2002 to 2015) [16]. In addition, it was reported that approximately 8% of non-cancer patients prescribed opioids after surgery became chronic users [17]. Considering the trends in opioid use in Korea, there is a need to evaluate opioid-related safety issues. However, there is a limitation to identifying the current status of ADEs of opioid analgesics in Korea, as studies reporting the safety of opioid analgesics use data from single-institution, judicial cases, or spontaneous ADE reports [18,19,20].

Based on prior literature, we aimed to evaluate the influence of opioid analgesic use patterns on all ED visits and opioid-related ED visits after opioid analgesic initiation using the national claims database.

## 2. Materials and Methods

### 2.1. Database and Population

We conducted a nested case–control study using the de-identified national claims database (2016–2018) provided by the Health Insurance Review Agency (HIRA). South Korea implements a national health insurance service covering approximately 98% of the population, and HIRA reviews the cost of medical care claimed by all healthcare institutes. Therefore, the database includes all healthcare utilization information regarding demographics, diagnoses, procedures performed, and prescription details.

All adults (≥18 years of age) without cancer, with the use of at least one non-injectable opioid analgesic (NIOA) between January 2017 and June 2018, were identified from the data source. NIOA include oral and transdermal opioid analgesics (Anatomical Therapeutic Chemical code: N02A and R05DA04) and exclude tramadol and combination agents for colds and coughs, because tramadol is not regulated as a controlled substance in Korea. The first NIOA claim during the above-mentioned period was defined as the cohort entry date. Patients with any record of cancer diagnosis during the study period were excluded (International Classification of Diseases (ICD)-10: C, D0, D32, D33, D37, D38, D39, and D4). We deliberately excluded patients with any NIOA claim record in the year preceding their cohort entry date, so that we could capture only those who were naïve to NIOA use. Patients were also excluded if they had received less than 7 days of continuous NIOA therapy, or if they did not have at least six months of follow-up data from their cohort entry date. 

We defined the case group as patients who visited the ED within six months after the cohort entry date. Only the first ED visit was considered for inclusion into the study when repeated ED visits occurred. The first date of the ED visit was defined as the index date. ED visits were divided into all-cause ED visits and opioid-related ED visits. Opioid-related ED visits, indicating ED visits associated with opioid-related adverse events (ORAE), were defined using the main diagnosis code or first sub-diagnosis code known to be related to opioid use as follows: (1) falls and fractures, (2) cardiovascular events, (3) respiratory depression, (4) dizziness, hypotension, and vertigo, (5) psychiatric (delirium, altered mental status, and opioid use disorder), and (6) others (nausea and vomiting, constipation and ileus, dry mouth, and urinary incontinence) (Table S1) [2]. In order to carry out an efficient analysis, the control group was randomly selected from among patients with an identical cohort entry year and follow-up period to the case group, in a 1:1 ratio. 

### 2.2. Definition of Variables

The main variable was the NIOA use pattern, as follows. First, the daily oral morphine milligram equivalents (MME), type of opioid formulation (immediate release (IR), sustained release (SR)), and number and type of NIOA were evaluated one month before the index date. Second, the status of NIOA use before the index date was classified as follows: none (no active NIOA prescriptions during −60 to −1 days), past user (active NIOA prescriptions only during −60 to −31 days), persistent user (active NIOA prescriptions during −60 to −1 days), and new user (active NIOA prescriptions only during −30 to −1 days). In addition, the cumulative duration of overall NIOA, number of NIOA prescriptions, and number of NIOA prescribers from initiation to event occurrence were identified. The indications and the department of opioid initiation were also considered based on the first NIOA prescription. Lastly, chronic use of NIOA was defined as more than 90 days of NIOA use during the six-month follow-up period. 

Participant sociodemographic characteristics (sex, age, and insurance), comorbidities, hospital utilization patterns before NIOA initiation, and co-medication one month before the index date were also identified as other variables. The categories of co-medications included benzodiazepines, gabapentinoids, z-drugs, tramadol, other pain medications, anti-platelet/anti-coagulants, digitalis, hypoglycemic agents, and anti-hypertensive agents. We also evaluated the anticholinergic burden and sedative load using the validated Korean anticholinergic burden scale and sedative load model [21,22]. To avoid duplicate evaluation among NIOA, anticholinergic burden, and sedative load, we did not allow evaluation overlap for each category.

### 2.3. Analysis

Descriptive statistics are expressed as the mean (standard deviation) and percentage, and the t-test and chi-square test were used to compare the case and control groups. We evaluated multicollinearity between variables and then adjusted the variables to be included in the multivariable logistic regression according to the clinical characteristics and variance inflation factors. We then performed univariate and multivariate logistic regression analyses to identify the association between NIOA use and ED visits. All variables, except multicollinearity and with α ≤ 0.1 in the univariate analysis, were included in the multivariate logistic regression. We conducted a subgroup analysis limited to ORAE ED visits. In addition, we performed a subgroup analysis to identify the association between opioid analgesic use and ED visits more clearly by eliminating patients with well-known factors causing ED visits (experience of ED visits) or without opioid analgesic use within 2 months before the index date. A subgroup analysis was conducted in patients without ED visits before NIOA initiation and in patients who had active NIOA prescriptions for 2 months before the index date. All statistical analyses were performed using SAS version 9.4 (SAS Institute Inc., Cary, NC, USA).

## 3. Results

### 3.1. Patient Characteristics

**Figure 1:**
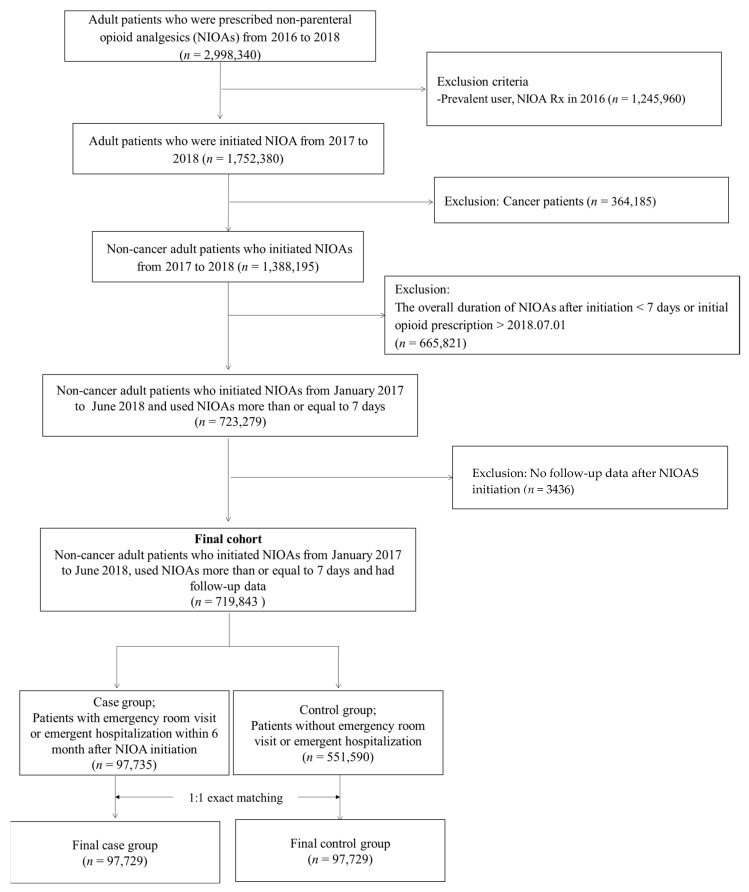
Patient selection flow. NIOA; non-injectable opioid analgesics.

A total of 2,998,340 adult patients were prescribed NIOA between 2016 and 2018. After exclusion of the non-eligible patients, such as prevalent users of NIOA, cancer patients, and patients with a limited duration of NIOA use (<seven days) or inappropriate follow-up periods for event identification, 719,843 (24.00%) patients were included. Among them, 97,735 (13.58%) patients visited the ED within six months. After 1:1 exact matching with non-ED visits, 195,458 patients were enrolled in the study (Figure 1).

The patients had a mean age of 60.25 ± 17.28 years, 43.21% were male, and 8.37% had Medicaid or National Meritorious Service (NMS) insurance. The most common comorbidities were hyperlipidemia (52.33%) and hypertension (48.25%), and the mean Charlson comorbidity index was 2.50 ± 2.26. Before NIOA initiation, almost one quarter of patients had visited the ED, and the mean number of days of healthcare utilization was 43.03 ± 49.64. Common co-medications used one month before the index date were other pain medications (73.61%) and anti-hypertensive agents (47.63%); 23.79% and 10.89% of the patients used benzodiazepines and gabapentinoids before the index date, respectively. The proportion of patients with a high daily anticholinergic burden (≥3) and sedative load (≥3) before the index date was 11.48% and 4.32%, respectively (Table 1).

### 3.2. NIOA Use Pattern

**Figure 2:**
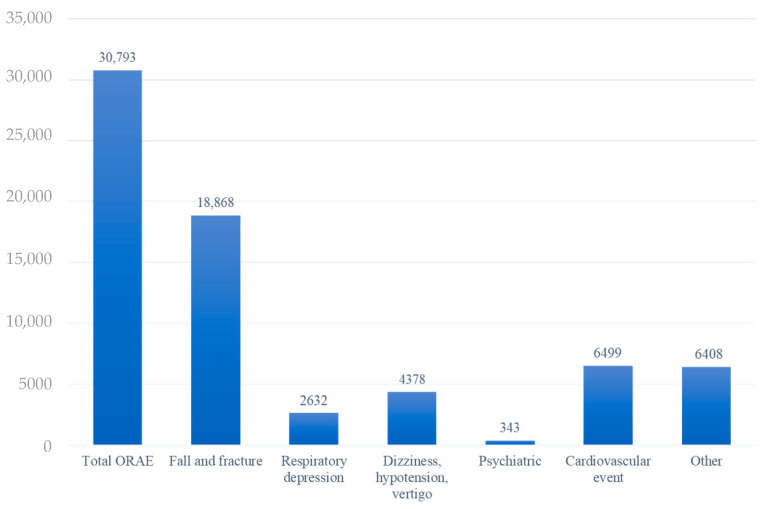
The number of opioid-related emergency department visits. Other: Nausea/vomiting, constipation, dry mouth, pruritus, urinary incontinence. Abbreviations: ORAE, opioid-related adverse drug events.

Approximately 40% of patients had NIOA initiated following surgery or trauma, and 16% of patients were first prescribed NIOA at the ED. Only 5.60% of patients were prescribed NIOA by more than two prescribers, and 2.8% of patients used NIOA for >90 days for six months after NIOA initiation. Approximately 60% of the patients used NIOA one month before the index date, and the daily MME of 2.6% was greater than 50. The most common ingredients in NIOA were codeine (21.72%) and buprenorphine (17.19%). ORAE ED visits were observed in 31.51% of the patients, with falls and fractures the most common type (61.27%) (Table 2 and Figure 2).

### 3.3. The Influence of NIOA Use Pattern on ED Visit

After adjusting for age, sex, insurance type, co-medications, comorbidities, and hospital utilization pattern before NIOA initiation among the factors for opioid use, indications other than surgery or trauma for opioid initiation (adjusted odds ratio (aOR), 1.20; 95% confidence interval (CI), 1.17–1.22), opioid initiation at ED (aOR, 3.19; 95% CI, 3.09–3.29), multiple prescribers for NIOA (aOR, 1.30; 95% CI, 1.24–1.36), chronic NIOA use (aOR, 1.32; 95% CI, 1.23–1.40), frequent NIOA prescription (aOR, 1.20; 95% CI 1.12–1.28), and high daily MME (≥50 MME/day, aOR, 1.20; 95% CI, 1.12–1.29) were associated with all-cause ED visits (Table 3).

The association between NIOA use patterns and ORAE ED visits showed a similar trend to all-cause ED visits. However, the risk of NIOA initiation at the ED and chronic NIOA use before the ED visit in ORAE ED visits was greater than that of all-cause ED visits (aOR, 3.82; 95% CI, 3.62–4.04; aOR, 1.56; 95% CI, 1.39–1.76, respectively). The risk of NIOA initiation at the ED was most significant in psychiatric events (aOR, 7.33; 95% CI, 3.46–15.52), and the risk of chronic NIOA use before the ED visits was greatest in patients with respiratory depression (aOR, 2.36; 95% CI, 1.53–3.63). Regarding fall and fracture events, the aORs of indications other than surgery or trauma for opioid initiation and high daily MME (≥50 MME/day) were significantly lower than those in the reference group (aOR, 0.81; 95% CI, 0.77–0.86; aOR, 0.76; 95% CI, 0.65–0.89; Table 3). 

The effects of other factors on all-cause and ORAE ED visits in the multivariate analysis are presented in Table S2. ED visits before NIOA initiation were the major factors influencing all-cause and ORAE ED visits after NIOA initiation. In the subgroup analysis of patients without ED visits before NIOA initiation and patients who had active NIOA prescriptions for two months before the index date, the effect of the NIOA use pattern was similar to that of all-cause and ORAE ED visits (Table S3).

## 4. Discussion

This nested case–control study of non-cancer patients with NIOA in South Korea between 2017 and 2018 found that 97,735 patients visited the ED within six months after NIOA initiation, accounting for 13% of all non-cancer patients who had NIOA for ≥seven days. Among them, we identified diagnosis codes related to opioid AEs in 32% of patients that visited the ED, accounting for 4% of all non-cancer patients who had NIOA for ≥seven days. After adjusting for age, sex, insurance type, healthcare utilization, comorbidities, and co-medications, the NIOA use pattern (opioid initiation site, number of NIOA prescribers, chronic NIOA use, and daily MME) was identified as a significant risk factor for all-cause or ORAE ED visits.

In the United States, which has the highest opioid consumption per capita, ~1000 per 100,000 patients have visited the ED annually because of prescribed opioids since 2016 [13]. A study conducted by Vivolo-Kantor et al. identified 142,557 (15.7 per 10,000 visits) opioid-related ED visits across the United States from July 2016 to September 2017 [23]. However, this could not be compared with our result because the previous study focused primarily on ED visits for overdose. In our study, the number of patients identified as having psychiatric AEs, including ICD-10 T-code, was only 343 (0.05%). Considering this discrepancy in the identified categories, ED visits in Korea could be lower than those in the United States. This finding is in line with a previous study in Korea, which reported that the incidence of opioid overdoses in NIOA incident users (0.05%) was lower than that in previous studies (0.4–0.6%) [24].

Our results showed that NIOA initiation due to chronic pain other than surgery or trauma is associated with an increased risk of opioid-related visits. This finding is not surprising, as opioid use for the long-term management of chronic pain in non-cancer patients is more likely to be associated with misuse and adverse effects than acute opioid use. Our recent study also confirmed that the major reason for potentially inappropriate opioid use in non-cancer patients is physical pain, which has a higher risk of opioid dependence and disorder due to the characteristics of persistent pain [25]. As per previous studies conducted in other countries, our results underscore the importance of opioid use for pain management in non-cancer patients with chronic physical pain.

Interestingly, our results indicated that patients on low-dose opioids (<50 MME) were less likely to have adverse effects and visit the ED. Opioids can play an essential role in pain management. However, there are questions regarding safe doses. Multiple guidelines have recommended that opioid prescriptions for non-cancer pain should not exceed 50 MME because of the risks associated with abuse and AEs. Schlosser et al. suggested that the opioid dose per day was the strongest factor in pain management [26]. They found that a low MME was associated with decreased readmission and length of hospital stay in patients undergoing hip and knee joint arthroplasty. Previous studies have shown that high doses of opioid analgesics are associated with an increased risk of opioid-related complications [27,28]. Our study results noted that low MME was not related to all-cause ED visits, including ORADE, falls, and fractures. An unexpected finding of our study was that even a high dose (>50 MME) was not associated with ORADE and was less likely to cause falls and fractures. However, caution is advised when interpreting these findings because the effect of the opioid dose changed to become non-significant in the multivariate analysis, different from the univariate analysis (OR 1.20, 95% CI 1.16~1.25). In other words, it could be interpreted that the effect of other opioid use patterns is more significant than that of the opioid dose.

Notably, our results showed that patients who initiated NIOA in the ED were more likely to visit the ED with all-cause or ORADE. After adjusting for the patient’s ED visit history, the results showed that patients who had NIOA initiated in the ED were at a high risk of another ED visit because of ADEs. In addition, the effect of NIOA usage patterns on ED visit occurrence in the subgroup analysis of patients without previous ED visits did not differ from that of all-cause ED visits or ORAE ED visits. These results suggest that intended exposure to opioids could drive an unintended prolonged pattern of use. Several studies have demonstrated an association between ED opioid initiation and the risk of long-term opioid use [29,30]. Hopped et al. previously found that opioid-naïve patients who had opioids initiated in the ED setting were at an increased risk of additional opioid use at one year [31]. Patients seeking help for uncontrolled pain are easily exposed to opioids in the ED because it is important to treat patients and relieve pain quickly and effectively. Given the higher likelihood of ED visits with AEs, our study results could serve as evidence to encourage the development of better opioid prescription guidelines and educational programs on opioid analgesic use in the ED.

Opioids have various adverse effects due to the widespread distribution of opioid receptors beyond the nervous system in the body. Some AEs can be fatal or lead to subsequent hospitalizations, including respiratory depression and cardiovascular events. In the present study, respiratory and cardiovascular events were identified at high rates. These AEs were strongly associated with chronic NIOA use. Most opioids have a minor negative effect on cardiac contractility. However, there can be significant changes in cardiac function when combined with other medications [32]. It is well known that the concomitant use of opioids and benzodiazepines results in a significantly higher rate of hospitalization and cardiac-related and respiratory mortality [33].

The population rates of ED visits differed according to age and insurance groups. Half of the population in the ED visit group was aged >65 years compared with those in the non-ED visit group (36.85%). This finding is explained by the fact that older patients are at a higher risk because of well-known risk factors, including comorbidities or polypharmacy [34]. A systematic review conducted by Megale et al. also demonstrated that the risk of AEs from opioids in older patients is three times higher than that in other age groups, with only minor benefits in pain management [35]. Several studies have identified that socioeconomic status is one of the primary determinants of substance dependence and subsequent ED visits across diseases [36,37]. Our results also showed two-fold higher rates of ED visits in patients with low socioeconomic status, beneficiaries of medical aid, and NMS. In addition, other studies have pointed out that chronic pain is a complex pathophysiological condition that is more likely to co-occur with other psychiatric comorbidities and the use of other substances. An earlier study found that United States veterans receiving high-dose opioid therapy were characterized by multiple pain problems and high levels of medical and psychiatric comorbidities [38]. Consistent with previous studies, we observed a higher population rate of psychiatric comorbidity and the use of other substances in the case groups.

This study has several limitations. First, this study carries the bias inherent to all studies using health insurance data, including inaccurate or lacking diagnostic documentation in claims data. The characteristics of claims data may lead to the underestimation or overestimation of the actual incidence of AEs. However, we only used primary or first sub-diagnosis codes to identify ORAE ED visits, to overcome these limitations. Moreover, due to the limited period of claims data, the follow-up period was too short to completely assess opioid-related adverse events. Second, we could not consider actual medication use as this study used a claims database. However, considering the agreement between actual medication use and medication adherence evaluation using claims data, this may not be a significant limitation. Third, we could not evaluate the clinical causality between opioid use and ED visits because of the nature of the claims data. However, we identified several studies that used diagnosis codes as indicators of ADEs or patient safety in claims data [39,40]. In the subgroup analysis of patients with an NIOA prescription for two months before the index date, in which the group had a temporally closer relationship between NIOA use and ED visits, we also confirmed that the association between the NIOA use pattern and ED visits did not differ from the primary analysis. Moreover, although the type of insurance as an indicator of economic or social status was used in the present study, we could not accurately evaluate the social level of the included patients because of the lack of direct information about the social status of beneficiaries in the claims database. Fourth, the specified classification of adverse events might have underestimated the results because some adverse events may be multifactorial. In addition, we could not estimate the severity of other adverse events or include fatal adverse events.

Due to the relatively low prevalence of opioid use in Korea, the value of hospital data for assessing opioid-related hospitalization or emergency visits might be limited. In this context, the first strength of our study is the use of an extensive nationwide insurance claims database that contains all ED and prescription records in Korea. In addition, considering the importance of ED visits as a surveillance method for opioid-related harm in other countries [41,42], this study could confirm the possibility of using ED data to monitor opioid-related events in Korea by identifying the association between NIOA use and ED visits.

## 5. Conclusions

In conclusion, this nationwide case–control study found that 13% of all non-cancer patients who initiated NIOA visited the ED within six months of NIOA initiation. In addition, the NIOA use pattern was significantly associated with an increased risk of both all-cause and opioid-related ED visits. Our study introduces a strong rationale for healthcare providers, researchers, and policymakers to continue their efforts to educate patients, conduct further studies, develop guidelines, and implement a multifaceted approach to facilitate appropriate opioid use.

## Figures and Tables

**Table 1:** Baseline characteristics of the patients who were initially prescribed NIOA for non-cancer pain.

	Total (*n* = 195,458)		Patients without ED Visit after NIOA Initiation (*n* = 97,729)		Patients with ED Visit after NIOA Initiation (*n* = 97,729)		*p*-Value
Variables	*n*	(%)	*n*	(%)	*n*	(%)	
Year of opioid initiation							
2017	132,772	(67.93)	66,386	(67.93)	66,386	(67.93)	1.000
2018	62,686	(32.07)	31,343	(32.07)	31,343	(32.07)	
Sex, Male	84,449	(43.21)	41,865	(42.84)	42,584	(43.57)	0.001
Age (years), mean ± SD	60.25 ± 17.28		57.58 ± 16.75		62.93 ± 17.39		<0.0001
≤55	67,171	(34.37)	38,652	(39.55)	28,519	(29.18)	<0.0001
55~≤65	42,557	(21.77)	23,064	(23.60)	19,493	(19.95)	
65~≤75	37,967	(19.42)	18,930	(19.37)	19,037	(19.48)	
75~≤85	36,629	(18.74)	13,796	(14.12)	22,833	(23.36)	
85~	11,134	(5.70)	3287	(3.36)	7847	(8.03)	
Insurance							
Medical insurance	179,089	(91.63)	92,328	(94.47)	86,761	(88.78)	<0.0001
Medicaid or NMS	16,369	(8.37)	5401	(5.53)	10,968	(11.22)	
Anxiety	47,188	(24.14)	18,476	(18.91)	28,712	(29.38)	<0.0001
Mood disorder	41,695	(21.33)	15,211	(15.56)	26,484	(27.10)	<0.0001
Other mental disorder	50,596	(25.89)	18,847	(19.28)	31,749	(32.49)	<0.0001
Substance use disorder	3000	(1.53)	619	(0.63)	2381	(2.44)	<0.0001
Hyperlipidemia	102,284	(52.33)	45,953	(47.02)	56,331	(57.64)	<0.0001
Hypertension	94,312	(48.25)	39,794	(40.72)	54,518	(55.78)	
Heart failure	19,941	(10.20)	5865	(6.00)	14,076	(14.40)	
Myocardial infarction	3595	(1.84)	1046	(1.07)	2549	(2.61)	<0.0001
Stroke	31,169	(15.95)	10,437	(10.68)	20,732	(21.21)	<0.0001
Diabetes mellitus	61,832	(31.63)	24,837	(25.41)	36,995	(37.85)	<0.0001
Respiratory disease	92,152	(47.15)	41,833	(42.81)	50,319	(51.49)	<0.0001
Severe renal disease	4979	(2.55)	1113	(1.14)	3866	(3.96)	<0.0001
Moderate to severe liver disease	1603	(0.82)	348	(0.36)	1255	(1.28)	<0.0001
Fall or fracture	95,355	(48.79)	43,027	(44.03)	52,328	(53.54)	<0.0001
Hypotension	2177	(1.11)	525	(0.54)	1652	(1.69)	<0.0001
Charlson comorbidity index, mean ± SD	2.50 ± 2.26		1.98 ± 1.89		3.03 ± 2.46		<0.0001
0	33,983	(17.39)	22,040	(22.55)	11,943	(12.22)	<0.0001
1	46,266	(23.67)	27,083	(27.71)	19,183	(19.63)	
2	37,046	(18.95)	19,030	(19.47)	18,016	(18.43)	
≥3	78,163	(39.99)	29,576	(30.26)	48,587	(49.72)	
Number of ED visits before NIOA initiation, mean ± SD	0.42 ± 1.32		0.19 ± 0.51		0.66 ± 1.76		<0.0001
0	146,985	(75.20)	83,321	(85.26)	63,664	(65.14)	<0.0001
1	32,539	(16.65)	11,701	(11.97)	20,838	(21.32)	
2	9273	(4.74)	2042	(2.09)	7231	(7.40)	
3	3300	(1.69)	447	(0.46)	2853	(2.92)	
≥4	3361	(1.72)	218	(0.22)	3143	(3.22)	
Days of healthcare utilization before NIOA initiation, mean ± SD	43.03 ± 49.64		34.19 ± 39.26		51.87 ± 56.83		<0.0001
≤21 days	71,896	(36.78)	44,247	(45.28)	27,649	(28.29)	<0.0001
22~≤65 days	87,810	(44.93)	41,396	(42.36)	46,414	(47.49)	
66~≤100	19,329	(9.89)	7283	(7.45)	12,046	(12.33)	
101~	16,423	(8.40)	4803	(4.91)	11,620	(11.89)	
Days of hospitalization before NOPA initiation, mean ± SD	16.4 ± 39.69		11.23 ± 30.49		21.57 ± 46.55		<0.0001
≤30	168,574	(86.25)	89,713	(91.80)	78,861	(80.69)	<0.0001
31~	26,884	(13.75)	8016	(8.20)	18,868	(19.31)	
Major type of hospital before NIOA initiation							
Clinic	162,157	(82.96)	84,332	(86.29)	77,825	(79.63)	<0.0001
Hospitals	23,369	(11.96)	9441	(9.66)	13,928	(14.25)	
Tertiary hospitals	9932	(5.08)	3956	(4.05)	5976	(6.11)	
Benzodiazepine	46,508	(23.79)	17,832	(18.25)	28,676	(29.34)	<0.0001
Gabapentinoids	21,276	(10.89)	8756	(8.96)	12,520	(12.81)	<0.0001
Z-drugs	12,733	(6.51)	4542	(4.65)	8191	(8.38)	<0.0001
Tramadol	81,314	(41.60)	34,754	(35.56)	46,560	(47.64)	<0.0001
Other pain medications	143,884	(73.61)	69,106	(70.71)	74,778	(76.52)	<0.0001
Anti-platelet or coagulants	57,700	(29.52)	21,896	(22.40)	35,804	(36.64)	<0.0001
Digitalis	2500	(1.28)	628	(0.64)	1872	(1.92)	<0.0001
Hypoglycemic agents	35,927	(18.38)	13,991	(14.32)	21,936	(22.45)	<0.0001
Anti-hypertensive agents	93,088	(47.63)	38,690	(39.59)	54,398	(55.66)	<0.0001
Daily anticholinergic burden, mean ± SD	1.28 ± 1.64		1.05 ± 1.39		1.51 ± 1.84		<0.0001
<3	173,011	(88.52)	89,415	(91.49)	83,596	(85.54)	<0.0001
≥3	22,447	(11.48)	8314	(8.51)	14,133	(14.46)	
Daily sedative load, mean ± SD	0.65 ± 1.30		0.53 ± 0.96		0.77 ± 1.55		<0.0001
<3	187,014	(95.68)	94,825	(97.03)	92,189	(94.33)	<0.0001
≥3	8444	(4.32)	2904	(2.97)	5540	(5.67)	

Abbreviations: ED, emergency department; NMS, National Meritorious Service; NIOA, non-injectable opioid analgesics; SD, standard deviation.

**Table 2:** Patterns of NIOA use in patients initially prescribed NIOA for non-cancer pain.

	Total (*n* = 195,458)		Patients without ED Visit after NIOA Initiation (*n* = 97,729)		Patients with ED Visit after NIOA Initiation (*n* = 97,729)		*p*-Value
Variables	*n*	(%)	*n*	(%)	*n*	(%)	
Indication of opioid initiation							
Surgery or trauma	79,051	(40.44)	36,942	(37.80)	42,109	(43.09)	<0.0001
Other	116,407	(59.56)	60,787	(62.20)	55,620	(56.91)	
Opioid initiation at the emergency department							
No	163,720	(83.76)	89,900	(91.99)	73,820	(75.54)	<0.0001
Yes	31,738	(16.24)	7829	(8.01)	23,909	(24.46)	
Number of NIOA prescribers	1.06 ± 0.26		1.05 ± 0.23		1.07 ± 0.28		<0.0001
1	184,511	(94.40)	93,364	(95.53)	91,147	(93.27)	<0.0001
2~	10,947	(5.60)	4365	(4.47)	6582	(6.73)	
Chronic NIOA use	20.84 ± 26.22		19.82 ± 23.70		21.86 ± 28.48		<0.0001
No	189,991	(97.20)	95,591	(97.81)	94,400	(96.59)	<0.0001
Yes	5467	(2.80)	2138	(2.19)	3329	(3.41)	
Number of NIOA prescriptions	1.54 ± 1.19		1.53 ± 1.12		1.56 ± 1.25		<0.0001
≤4	189,913	(97.16)	95,347	(97.56)	94,566	(96.76)	<0.0001
5~	5545	(2.84)	2382	(2.44)	3163	(3.24)	
Number of NIOA	0.62 ± 0.61		0.58 ± 0.59		0.65 ± 0.63		<0.0001
0	87,013	(44.52)	45,332	(46.39)	41,681	(42.65)	<0.0001
1	97,589	(49.93)	48,189	(49.31)	49,400	(50.55)	
2	9880	(5.05)	3861	(3.95)	6019	(6.16)	
≥3	976	(0.5)	347	(0.36)	629	(0.64)	
Daily MME	8.45 ± 15.36		6.76 ± 12.09		10.15 ± 17.89		<0.0001
0	87,013	(44.52)	45,332	(46.39)	41,681	(42.65)	<0.0001
0~≤50	103,451	(52.93)	51,070	(52.26)	52,381	(53.6)	
50~	4994	(2.56)	1327	(1.36)	3667	(3.75)	
NIOA use pattern							
None	59,562	(30.47)	31,057	(31.78)	28,505	(29.17)	<0.0001
Past user	27,451	(14.04)	14,275	(14.61)	13,176	(13.48)	
Persistent user	35,353	(18.09)	16,162	(16.54)	19,191	(19.64)	
New user	73,092	(37.4)	36,235	(37.08)	36,857	(37.71)	
Formulation of NIOA							
None	87,013	(44.52)	45,332	(46.39)	41,681	(42.65)	<0.0001
Immediate release	47,812	(24.46)	25,846	(26.45)	21,966	(22.48)	
Sustained release	60,633	(31.02)	26,551	(27.17)	34,082	(34.87)	
Type of NIOA							
None	87,013	(44.52)	45,332	(46.39)	41,681	(42.65)	<0.0001
Buprenorphine	33,591	(17.19)	16,794	(17.18)	16,797	(17.19)	
Codeine	42,444	(21.72)	23,561	(24.11)	18,883	(19.32)	
Dihydrocodeine	671	(0.34)	256	(0.26)	415	(0.42)	
Fentanyl	8424	(4.31)	2262	(2.31)	6162	(6.31)	
Hydrocodone	1371	(0.7)	729	(0.75)	642	(0.66)	
Hydromorphone	2273	(1.16)	983	(1.01)	1290	(1.32)	
Morphine	476	(0.24)	257	(0.26)	219	(0.22)	
Oxycodone	7675	(3.93)	3105	(3.18)	4570	(4.68)	
Tapentadol	664	(0.34)	242	(0.25)	422	(0.43)	
≥2	10,856	(5.55)	4208	(4.31)	6648	(6.80)	

Abbreviations: ED emergency department; MME, oral morphine milligram equivalents; NIOA, non-injectable opioid analgesics; ORAE, opioid-related adverse drug events.

**Table 3:** Association between the patterns of NIOA use and ED visits after NIOA initiation.

	All-Cause ED Visit		ORAE ED Visit		Fall and Fracture		Cardiovascular Event		Respiratory Depression		Dizziness, Hypotension, Vertigo		Others ^(1)^		Psychiatric	
	(*n* = 195,458)		(*n* = 61,586)		(*n* = 37,736)		(*n* = 12,998)		(*n* = 5264)		(*n* = 8756)		(*n* = 12,816)		(*n* = 686)	
	aOR	(95% CI)	aOR	95% CI	aOR	95% CI	aOR	95% CI	aOR	95% CI	aOR	95% CI	aOR	95% CI	aOR	95% CI
Indication of opioid initiation																
Surgery or trauma	1		1		1		1		1		1		1		1	
Other	1.2	(1.17~1.22)	1.01	(0.97~1.05)	0.81	(0.77~0.86)	1.21	(1.09~1.34)	1.29	(1.12~1.49)	1.46	(1.32~1.62)	1.29	(1.19~1.41)	0.96	(0.58~1.58)
Opioid initiation at the emergency department																
No	1		1		1		1		1		1		1		1	
Yes	3.19	(3.09~3.29)	3.82	(3.62~4.04)	4.91	(4.58~5.27)	3.31	(2.9~3.78)	2.55	(2.1~3.08)	1.35	(1.14~1.61)	1.77	(1.56~2.02)	7.33	(3.46~15.52)
Number of NIOA prescribers																
1	1		1		1		1		1		1		1		1	
2~	1.3	(1.24~1.36)	1.19	(1.1~1.29)	1.09	(0.97~1.22)	1.2	(0.98~1.47)	1.23	(0.94~1.62)	1.23	(1.01~1.51)	1.49	(1.26~1.75)	1.64	(0.53~5.05)
Chronic NIOA use																
No	1		1		1		1		1		1		1		1	
Yes	1.32	(1.23~1.40)	1.56	(1.39~1.76)	1.61	(1.37~1.9)	1.48	(1.09~2.00)	2.36	(1.53~3.63)	1.44	(1.09~1.92)	1.4	(1.11~1.77)	2.84	(0.82~9.9)
Number of NIOA prescriptions																
≤4	1		1		1		1		1		1		1		1	
5~	1.2	(1.12~1.28)	1.34	(1.19~1.51)	1.53	(1.3~1.8)	2.14	(1.56~2.93)	1.57	(1.04~2.35)	1.08	(0.82~1.42)	0.99	(0.79~1.25)	4.17	(0.9~19.35)
Daily MME																
0	1		1		1		1		1		1		1		1	
0~≤50	0.82	(0.8~0.84)	0.75	(0.72~0.78)	0.55	(0.52~0.58)	0.62	(0.56~0.69)	0.95	(0.82~1.09)	1.04	(0.94~1.15)	1.01	(0.93~1.1)	0.73	(0.43~1.22)
50~	1.2	(1.12~1.29)	0.97	(0.86~1.11)	0.76	(0.65~0.89)	0.99	(0.74~1.34)	1.03	(0.67~1.59)	1.18	(0.79~1.76)	1.23	(0.9~1.68)	0.52	(0.16~1.7)

^(1)^ Others: nausea/vomiting, constipation, dry mouth, pruritus, urinary incontinence. In the multivariate analysis, age, sex, insurance type, co-medications, comorbidities, and the hospital utilization pattern before NIOA initiation were adjusted. Abbreviations: aOR, adjusted odds ratio; ED, emergency department; NMS, National Meritorious Service; NIOA, non-injectable opioid analgesics.

## Data Availability

Data were obtained from the Health Insurance Review Agency and are available at https://opendata.hira.or.kr/home.do (accessed on 3 June 2022), with the permission of the Health Insurance Review Agency.

## Supplementary Materials

The following supporting information can be downloaded at: https://www.mdpi.com/article/10.3390/medicina59030519/s1, Table S1. ICD-10 codes for comorbidity identification. Table S2. Multivariate analysis for all-cause and ORAE ED visits after NIOA initiation. Table S3. Subgroup analysis in patients without ED visits before NIOA initiation or in patients who used NIOA for 2 months before the index date.
