# Supplementary material for: Risk Factors for Emergency Department Presentations after the Initiation of Opioid Analgesics in Non-Cancer Patients in Korea: A Nationwide Study
Source: Medicina (Kaunas). 2023 Mar 7;59(3):519. doi: 10.3390/medicina59030519 (PMC10056559; doi:10.3390/medicina59030519)
Supplement: Supplementary file 1 [file medicina-59-00519-s001.zip › medicina-2179308-supplementary.pdf]

Table S1. ICD-10 codes for comorbidities identification

Category	ICD-10 codes
**Anxiety**	'F064', 'F068', 'F40', 'F41', 'F42', 'F43', 'F452', 'F633', 'F930', 'F938', 'F940'
**Mood disorder**	'F063', 'F301', 'F302', 'F308', 'F31', 'F32', 'F33', 'F34', 'F38', 'F39'
**Substance abuse**	'F10', 'F11', 'F12', 'F13', 'F14', 'F15', 'F16', 'F17', 'F18', 'F19', 'F55'
**Other mental disorder**	F code excluding anxiety, mood disorder, substance abuse
**Hyperlipidemia**	E78
**Hypertension**	'I10', 'I11', 'I12', 'I13','I15
**Heart failure**	I50', 'I110', 'I130', 'I132'
**Myocardial infarction**	'I21', 'I22', 'I23
**Stroke**	'I6', 'G45', 'G46'
**Diabetes mellitus**	E10', 'E11', 'E12', 'E13', 'E14
**Respiratory disease**	I278', 'I279', 'J4', 'J60', 'J61', 'J62', 'J63', 'J64', 'J65', 'J66', 'J67', 'J684', 'J701', 'J703
**Severe Renal disease**	'I120', 'I1311', 'I132', 'N185', 'N186', 'N19', 'N250', 'Z49', 'Z992
**Moderate to severe liver disease**	I85', 'K704', 'K72','K766', 'B150', 'B160', 'B162', 'B190'
**Fall or fracture**	M80', 'M84', 'M90', 'S01', 'S02', 'S051', 'S059', 'S06', 'S097', 'S098', 'S099', 'S109', 'S119', 'S12', 'S13', 'S141', 'S200', 'S202', 'S211', 'S212', 'S22','S231', 'S232', 'S233', 'S234', 'S235', 'S241', 'S290', 'S32', 'S400', 'S410', 'S410', 'S42', 'S43', 'S460', 'S461', 'S468', 'S500', 'S501', 'S510', 'S52', 'S600', 'S601', 'S602', 'S610', 'S62', 'S63', 'S669', 'S700', 'S701', 'S72','S82', 'S83', 'S86', 'S900', 'S901', 'S902', 'S903', 'S910', 'S911', 'S913', 'S92', 'T02', 'T07', 'T08', 'T10', 'T12', 'T142', 'T148', 'T149', 'T902', 'T911', 'T912', 'T921', 'W0', 'W1'
**Hypotension**	'I95'
**Dizziness**	'R42'
**Delirium**	'F05'
**Constipation, ileus**	'K590', 'K56'
**Nausea/Vomiting**	'R11'
**Urinary incontinence**	'R33', 'R391', 'N40'
**Altered mentality**	'R400', 'R410', 'R418', 'R401'
**Vertigo**	'H814'
**Pruritus**	'L299'
**Respiratory depression**	'J96', 'J80', 'R060', 'R068'
**Dry mouth**	'R682'
**Opioid use disorder**	F11, T40

Abbreviation: ICD, International Classification of Diseases

Table S2. Multivariate analysis for all-cause and ORAE ED visit after NIOA initiation.

Variables	All cause ED visit		ORAE ED visit	
	aOR	95% CI	aOR	95% CI
**Sex,** Male	1		1	
Female	0.92	(0.9-0.94)	0.92	(0.88~0.95)
**Age**				
≤ 55years	1		1	
55~≤65	0.94	(0.92~0.97)	1.06	(1~1.11)
65~≤75	0.95	(0.92~0.98)	1.13	(1.07~1.2)
75~≤85	1.31	(1.26~1.35)	1.76	(1.66~1.87)
85~	1.69	(1.61~1.78)	2.52	(2.3~2.76)
**Insurance,** Medical insurance	1		1	
Medicaid or NMS	1.23	(1.19~1.28)	1.25	(1.17~1.34)
**Anxiety**	1.09	(1.07~1.12)	1.11	(1.06~1.16)
**Mood disorder**	1.13	(1.1~1.16)	1.22	(1.16~1.28)
**Other mental disorder**	1.09	(1.06~1.12)	1.12	(1.07~1.17)
**Substance abuse**	1.66	(1.5~1.83)	1.93	(1.61~2.32)
**Hyperlipidemia**	0.98	(0.96~1.01)	0.98	(0.94~1.02)
**Heart failure**	1.21	(1.17~1.26)	1.07	(1~1.15)
**Myocardial infarction**	1.13	(1.04~1.22)	0.99	(0.85~1.15)
**Stroke**	1.18	(1.14~1.22)	1.14	(1.07~1.2)
**Respiratory disease**	1.15	(1.12~1.17)	1.07	(1.03~1.11)
**Severe Renal disease**	1.66	(1.54~1.78)	1.36	(1.17~1.57)
**Moderate to severe liver disease**	1.66	(1.46~1.9)	1.32	(1.03~1.7)
**Fall or fracture**	1.1	(1.08~1.13)	1.56	(1.5~1.62)
**Hypotension**	1.21	(1.08~1.35)	1.41	(1.15~1.72)
**Number of ED visits before NIOA initiation**				
0	1		1	
1	1.88	(1.83~1.93)	1.73	(1.65~1.81)
2	3.16	(3~3.34)	2.79	(2.53~3.08)
3	4.88	(4.4~5.42)	3.6	(3~4.33)
≥ 4	9.67	(8.4~11.14)	7.61	(5.88~9.86)
**Days of healthcare utilization before NIOA initiation**				
≤ 21days	1		1	
22~≤65 days	1.15	(1.12~1.18)	1.06	(1.01~1.1)
66~≤100	1.19	(1.14~1.24)	1.07	(0.99~1.15)
101~	1.26	(1.2~1.32)	1.11	(1.01~1.21)
**Days of hospitalization before NIOA initiation**				
≤ 30	1		1	
31~	1.13	(1.09~1.17)	1.02	(0.95~1.08)
**Major type of hospital before NIOA initiation**				
Clinic	1		1	
Hospitals	1.31	(1.26~1.35)	1.31	(1.24~1.39)
Tertiary hospitals	1.48	(1.42~1.55)	1.27	(1.17~1.39)
**Benzodiazepine**	1.19	(1.16~1.22)	1.15	(1.1~1.2)
**Gabapentinoids**	1.1	(1.06~1.13)	0.99	(0.94~1.05)
**Z-drugs**	1.22	(1.2~1.25)	1.08	(0.99~1.19)

**Tramadol**	1.22	(1.2~1.25)	1.24	(1.18~1.29)
**Other pain medication**	1.15	(1.12~1.19)	1.1	(1.05~1.15)
**Anti-platelet or coagulants**	1.12	(1.09~1.15)	1.06	(1.01~1.11)
**Digitalis**	1.23	(1.12~1.37)	1.13	(0.93~1.38)
**Hypoglycemic agents**	1.13	(1.1~1.16)	1.07	(1.02~1.13)
**Anti-hypertensive agents**	1.13	(1.1-1.15)	1.07	(1.03~1.12)
**Daily anticholinergic burden**				
<3	1		1	
≥ 3	1.2	(1.16~1.24)	1.21	(1.14~1.28)
**Daily sedative load**				
<3	1		1	
≥ 3	1.09	(1.03~1.16)	1.16	(1.03~1.3)
**Indication of opioid initiation**				
Surgery or trauma	1		1	
Other	1.2	(1.17~1.22)	1.01	(0.97~1.05)
**Opioid initiation at the emergency department**				
No	1		1	
Yes	3.19	(3.09~3.29)	3.82	(3.62~4.04)
**Number of NIOA prescribers**				
1	1		1	
2~	1.3	(1.24-1.36)	1.19	(1.1~1.29)
**Chronic NIOA use**				
No	1		1	
Yes	1.32	(1.23~1.4)	1.56	(1.39~1.76)
**Number of NIOA prescriptions**				
≤ 4	1		1	
5~	1.2	(1.12~1.28)	1.34	(1.19~1.51)
**Daily MME**				
0	1		1	
0~≤50	0.82	(0.8~0.84)	0.75	(0.72~0.78)
50~	1.2	(1.12~1.29)	0.97	(0.86~1.11)

Abbreviation: aOR, adjusted odds ratio; ED, emergency department; NMS, National Meritorious Service; NIOA, non-parenteral opioid analgesics; ORAE, opioid-related adverse drug events

Table S3. Subgroup analysis in patients without ED visit before NIOA initiation or in patients who used NIOA for 2 months before the index date

	**No ED visit before NIOA initiation** (N=146,985)		**NIOA user 2 months before the index date** (N=135,896)	
	aOR	(95% CI)	aOR	(95% CI)
**Indication of opioid initiation**				
Surgery or trauma	1		1	
Other	1.2	(1.17-1.23)	1.17	(1.14-1.21)
**Opioid initiation at the emergency department**				
No	1		1	
Yes	3.51	(3.39-3.64)	4.09	(3.94-4.24)
**Number of NIOA prescribers**				
1	1		1	
2~	1.33	(1.26-1.40)	1.37	(1.31-1.45)
**Chronic NIOA use**				
No	1		1	
Yes	1.34	(1.24-1.44)	1.31	(1.23~1.4)
**Number of NIOA prescriptions**				
≤ 4	1		1	
5~	1.2	(1.12~1.29)	1.29	(1.2~1.38)
**Daily MME 1month**				
0	1		1	
0~≤50	0.81	(0.79-0.83)	0.92	(0.89-0.95)
50~	1.27	(1.17-1.38)	1.29	(1.19-1.39)

Abbreviation: aOR, adjusted odds ratio; ED, emergency department; NIOA, non-parenteral opioid analgesics; NMS, National Meritorious Service
